# Smart Multi-Level Tool for Remote Patient Monitoring Based on a Wireless Sensor Network and Mobile Augmented Reality

**DOI:** 10.3390/s140917212

**Published:** 2014-09-16

**Authors:** Fernando Cornelio Jimènez González, Osslan Osiris Vergara Villegas, Dulce Esperanza Torres Ramírez, Vianey Guadalupe Cruz Sánchez, Humberto Ochoa Domínguez

**Affiliations:** 1 Departamento de Ingeniería, Universidad Tecnológica de Ciudad Juárez, Av. Universidad Tecnológica 3051, Ciudad Juárez, Chihuahua, Mexico; E-Mail: dulce_torres@utcj.edu.mx; 2 Instituto de Ingeniería y Tecnología, Universidad Autónoma de Ciudad Juárez, Av. del Charro, 450 norte, Ciudad Juárez, Chihuahua, Mexico; E-Mails: overgara@uacj.mx (O.O.V.V.); vianey.cruz@uacj.mx (V.G.C.S.); hochoa@uacj.mx (H.O.D.)

**Keywords:** wireless sensor network, mobile augmented reality, remote patient monitoring, temperature sensor, heart rate sensor, Arduino, Android OS

## Abstract

Technological innovations in the field of disease prevention and maintenance of patient health have enabled the evolution of fields such as monitoring systems. One of the main advances is the development of real-time monitors that use intelligent and wireless communication technology. In this paper, a system is presented for the remote monitoring of the body temperature and heart rate of a patient by means of a wireless sensor network (WSN) and mobile augmented reality (MAR). The combination of a WSN and MAR provides a novel alternative to remotely measure body temperature and heart rate in real time during patient care. The system is composed of (1) hardware such as Arduino microcontrollers (in the patient nodes), personal computers (for the nurse server), smartphones (for the mobile nurse monitor and the virtual patient file) and sensors (to measure body temperature and heart rate), (2) a network layer using WiFly technology, and (3) software such as LabView, Android SDK, and DroidAR. The results obtained from tests show that the system can perform effectively within a range of 20 m and requires ten minutes to stabilize the temperature sensor to detect hyperthermia, hypothermia or normal body temperature conditions. Additionally, the heart rate sensor can detect conditions of tachycardia and bradycardia.

## 1. Introduction

Because of changes in technology, a continuous evolution is occurring in industries such as medical services, industrial manufacturing, and comfort services. In these industries, technological advances must be monitored to support decisions or actions that could benefit the processes supporting the industry. In medical services, researchers are developing teleoperated robots, prostheses, and smart monitoring systems to benefit services such as medical interventions, medical studies, and medical care.

In public health institutions (PHIs) in some countries, the excessive number of hospitalized patients cared for by the nursing staff represents a problem [1]. The nurses invest substantial amounts of time updating information about each patient's health by measuring values such as body temperature, heart rate, and glucose levels.

Nurses play an important role in caring for the sick, because they are in direct contact with the patient when taking vital signs. Often, nurses and doctors record information about their patients' progress by filling out reports by hand. A smart monitoring system could measure the patients' progress in real time, providing continuous feedback on the improvement in the health of patients who have undergone treatment. Additionally, nurses and doctors could save valuable response time with real-time notifications. Integrating intelligent monitoring systems could improve care by providing the nurses with efficient documentation and timely access to information [2]. The remote monitoring of vital signs could be useful in overcrowded PHIs, especially for the continuous monitoring of patients in the emergency department or of unattended patients when there is concern that the condition of a patient in the waiting area could deteriorate suddenly without being observed [3]. WSNs are being used to provide feedback during the monitoring process. WSNs can be used to support intelligent decisions, and moreover, these networks can provide data from many process points, providing an overall view of conditions [4]. However, if an image is better than a thousand words, handwritten reports produced by nurses and physicians could be replaced by an MAR environment used as a tool to facilitate the management of the patient information obtained from a WSN.

In this paper, a real-time smart multi-level tool for remote patient monitoring (SMTRPM) is proposed that integrates two cutting-edge technologies, WSN and MAR, to support nurses and physicians in PHIs. The SMTRPM uses WiFi technology to transmit data, reports, and alarms to a nurse server or mobile device in wireless mode. Moreover, smart nodes evaluate the patient's condition through data obtained from biometric sensors (body temperature and heart rate). Finally, virtual files implemented as an Android application complement handwritten nursing reports. The rest of this paper is organized as follows. Section 2 presents related research. In Section 3, a description of the novel SMTRPM is presented. Test results are presented in Section 4. Finally, Section 5 concludes the paper.

## 2. Related Work

The rapid development of wireless communication protocols has enabled the development of wireless medical devices [5,6]. Wireless capability refers to the transmission of biomedical information obtained from sensors or embedded systems through a wireless communication channel to a remote medical station or mobile device. In the literature, many efforts have been made to advance this capability. For example, He *et al.* [7] have developed a wireless technique to transmit information between biometric sensors and a monitoring center to increase the free space surrounding the patient. Additionally, the system, which is based on ZigBee technology, aims to improve the management of hospital services. The monitoring terminal can detect the patient's body temperature, heart rate and other physiological information in real time and transmit this information to the control center.

Fensli *et al.* [8] present a new concept for wireless electrocardiogram (ECG) monitoring, specially designed for arrhythmia diagnostics, based on a smart electrode with wireless transmission capability. The system acts as a continuous event recorder that can be used to monitor patients with arrhythmia.

Misra *et al.* [9] introduce a multi-tier telemedicine system to perform real-time analysis of sensor data. Several intelligent devices are integrated into a wireless body area network (WBAN) to create a system for health monitoring. The system performs real-time analysis of sensor data. In addition, it uses cloud storage so that data can be retrieved at any time anywhere.

Table 1 lists some of the works that have been performed on wireless patient monitoring systems. Each research project attempts to solve a specific problem and uses different technologies, such as different types of sensors, monitors, and wireless communication technology.

Remote patient monitoring faces several obstacles. Older patients might be unfamiliar with the technology, and people of all ages might still have to be persuaded to use it. A wearable device could save lives. For example, smart watches can be used to manage chronic problems and optimize fitness programs. Wearable devices can provide output and connect to the Internet in various ways. Some devices enable wearers to monitor their own data using a mobile phone and a special website. Other devices allow data to be downloaded and viewed by third parties such as healthcare managers or clinicians watching for alarming trends that call for medical intervention.

Hospital patients are often tethered to various monitors, pumps, and medical gear. A brief review of commercial devices is presented here.

The IntelliVue MX40 from Philips Electronics attempts to simplify patient monitoring by integrating telemetry into a compact wearable patient monitor that can be used to monitor ambulatory patients and patients during transport. The device helps to save nurses' time because it allows them to check their patients' ECG rhythms without calling a technician at a central monitoring station.

Numera is developing a soon-to-be-released wearable mobile device that will provide two-way hands-free voice communication through a cellular network, GPS location tracking, and automated fall detection algorithms for personal emergency response services (PERS). Numera devices will also be equipped with telehealth gateway technology, allowing patients to upload biometric measurements from a variety of health devices through the mobile personal health gateway, to receive personalized reminders to take medications, to upload measurements, and to receive coaching specific to their health conditions.

Zephyr Technology's BioHarness BT sensor technology is used by third-party manufacturers of wearable fitness gear to add biometric monitoring capabilities. An example of this technology is under Armour's E39 electronic compression garment, which tracks data such as the wearer's breathing and heart rate. The data can be transmitted to a computer or mobile device. Other measures that the BioHarness BT can monitor are blood oxygen, ECG, and blood pressure. The device can also connect to a smartphone to transmit data to the Zephyr portal. From there, the data can be pushed to the user's personal electronic health record or to dispatch and service center applications on the World Wide Web.

The Nike FuelBand, a fitness monitoring wristband, measures and displays four metrics: time, calories, steps, and “NikeFuel,” a metric that measures the user's physical activity. NikeFuel is a proprietary technology that measures activity through the movement of the user's wrist and uses algorithms based on oxygen kinetics. Unlike calorie counts, which vary based on gender and body type, NikeFuel is “a normalized score that awards all participants equal scoring for the same activity regardless of their physical makeup”. Nike FuelBand users can also choose to receive a calorie count to compare how many calories have been burned with how much NikeFuel has been earned.

The main disadvantage of these devices is their price. Another disadvantage is that generally, these commercial products do not provide open source for development. Additionally, although there are applications of wireless biomedical monitoring systems, the literature review exposes some areas of opportunity that have not been explored at all, such as the integration of *ad hoc* wireless networks, the development of applications for mobile devices (*i.e.*, smartphones), the use of augmented reality and the performance of analyses for basic diagnostics. The SMTRPM exploits these opportunity areas in the design of a new open alternative for real-time remote patient monitoring.

## 3. SMTRPM Design

**Figure 1.**
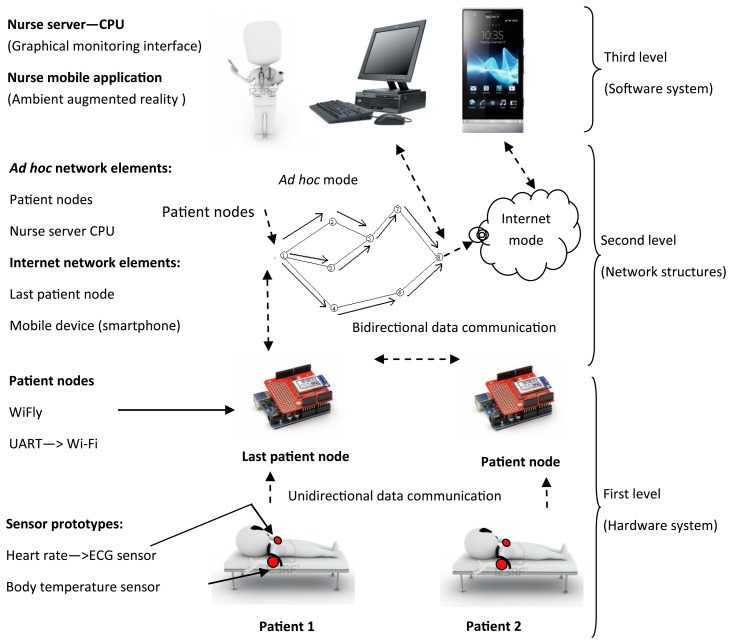
General schematic of the levels of the SMTRPM.

The SMTRPM provides personalized, intelligent, graphic, non-invasive, and real-time patient health monitoring. The SMTRPM is based on single patient nodes, a nursing server, and an Android based smartphone connected to a WiFi network. The overall architecture is divided into three main levels: (1) the hardware system (sensors, smart nodes, and wireless capability); (2) network structures (*ad hoc* and Internet modes); and (3) the software system (the nurse server interface, mobile nurse monitor and virtual patient file). Figure 1 presents a general schematic of the SMTRPM.

A patient node is assigned to each patient in a hospital room. Each patient node can communicate with other patient nodes in the network. The last patient node is configured to send data to the nurse server (a PC in the central nursing station) and to a smartphone (running an Android application) carried by the nurse. The specific characteristics of the hardware system, network structures, and software system are described in the following subsections.

### 3.1. Hardware System

The patient node is the most important entity in the hardware system. The patient node can sense, sample, and process one or more biometric signals. For this research, the initial hardware system includes prototype sensors to sense body temperature (BT) and heart rate (HR). These two variables are initialized manually by the nurse. The prototype SMTRPM measures only the BT and HR to demonstrate the concept of smart remote monitoring. However, more sensors could be added.

Each patient node is based on an Arduino Uno, which is an open source microcontroller platform for electronic prototyping that provides a flexible interface between hardware and software [15]. The Arduino Uno platform is used to acquire, process, and transmit the biometric signals. Additionally, a WiFly shield (roving networks) is incorporated into each Arduino to provide WiFi communication; this capability is necessary for communication with other patient nodes, the nurse server, and the smartphone.

**Figure 2.**
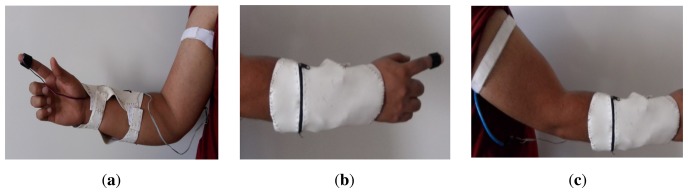
Sink node connected to BT and HR sensors: (**a**) general view of the sink node; (**b**) sink node attached to HR sensor; (**c**) sink node attached to BT sensor.

The SMTRPM is a prototype that is expected to be used for hospitalized patients in PHIs. It is assumed that the patients' mobility within their rooms will be limited. The sink node (the Arduino Uno with the WiFly shield) is attached to the patient through a flexible band attached to the forearm, as shown in Figure 2. The sink node must be placed inside an antistatic bag and has the capability to operate with external 9 V batteries. Therefore, the SMTRPM allows the patient to be mobile within a room.

#### 3.1.1. Design of Sensor Prototypes

Each patient node monitors the activity of two sensors, the BT sensor and the HR sensor, and informs other patient nodes about any events. The design of the sensor prototypes is explained in the following.

**Figure 3.**
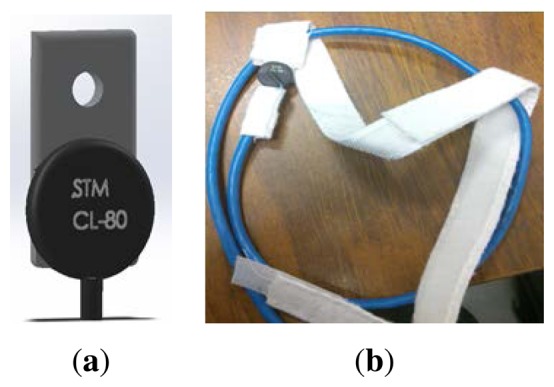
BT prototype: (**a**) BT sensor based on an NTC-CL80 thermistor and (**b**) elastic band with the thermistor located to acquire the patient's BT.

**Figure 4.**
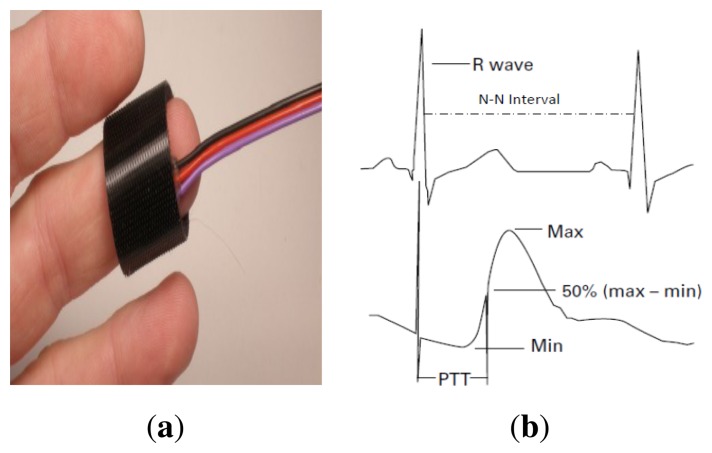
Heart rate prototype: (**a**) the photoplethysmographic sensor located on the patient's finger and (**b**) an example of an ECG curve showing how to identify the R wave and the N-N interval.

(a) Body Temperature Sensor Prototype
  The BT is a critical measure of the health status of the patient. Typically, the BT can be classified into three categories: hypothermia, which occurs when the patient temperature is below 36.0 °C; normothermia, which is the acceptable range of 37.5 °C to 36.5 °C for the human body; and hyperthermia, which occurs when the patient temperature exceeds 37.5 °C. Hypothermia is considered a potentially fatal emergency condition, and it is possible that lethargy, cardiac arrest, or even coma could occur if it is not treated quickly. Hyperthermia is an elevated body temperature due to failed thermoregulation, a situation that may cause organ damage or death [16].
  There are many places where the BT may be measured, including the armpit, mouth, rectum, and ears. In general, the armpit is the easiest location to measure the BT, and nurses place a thermometer under the patient's armpit to acquire the BT. For this reason, an elastic band was developed to locate the sensor in the armpit. The prototype for BT measurement was developed for the purpose of testing the SMTRPM; consequently, the medical security protocols that must be followed when testing medical devices in PHIs were not incorporated in the design. The final BT prototype is shown in Figure 3. The prototype is an assembly of an NTC-CL80 thermistor and an elastic band. When the device is placed on the patient, the sensor must be located in contact with the patient's armpit.
  Conventional digital thermometers use different types of thermistors to acquire the body temperature. The BT prototype was characterized using Equation (1), based on the NTC-CL80 thermistor curve [17].
  (1) $$K=(\beta )\left(\frac{1}{\text{In}\left(\frac{R}{R_{0}}\right)+\left(\frac{\beta }{T_{0}}\right)}\right)$$
  where *β* is the constant of the thermistor material, *R_0_* and *T_0_* are reference points on the NTC thermistor characterization curve, *R* is the resistance value of the body temperature, *In* is the natural logarithm, and *K* is the BT of the patient in degrees Kelvin.
  The BT prototype was tested and compared with a commercial thermometer using different *β* values. Note that *β* is temperature dependent and is specified between two temperature points on the NTC-CL80 curve. Different *β* values were compared in order to improve the accuracy of the BT sensor. The body temperature of the test patient was 36.5 °C when measured with a digital thermometer. The closest match between the instruments was obtained for *β* = 3260.5, with a relative error value of 0.001. This *β* value was used in the program implementing the BT algorithm, which is described in Section 3.1.2. Table 2 shows the experimental results from the 30 comparative runs between the BT prototype and the commercial thermometer, which were performed for three values of *β*.
(b) Heart Rate Sensor Prototype
  The HR is one of the most critical measures of patient health. In fact, evidence of high HR variability can support diagnoses such as infection, high levels of triglycerides or cholesterol, and lethal arrhythmias [18,19]. The SMTRPM is able to measure the patient's HR through a non-invasive photoplethysmographic sensor shown in Figure 4a. The system detects the heart beats per minute (BPM) and normal-to-normal heartbeat intervals (N-N intervals) from a continuous ECG wave record. Conventionally, the time at which the photoplethysmographic pulse waveform reaches 50% of its maximum value is used to indicate the beginning of a pulse [20]. The N-N interval is the elapsed time between pulses. Figure 4b shows the R wave and the corresponding N-N interval.
  The SMTRPM system uses the BPM information to determine the category of the heart rate: normal (60–100 BPM), tachycardia (ϯ100 BPM) [21], or bradycardia (<60 BPM) [22]. In some cases, tachycardia can significantly disrupt normal heart function, increasing the risk of stroke or causing sudden cardiac arrest or death. Bradycardia can be a serious problem if the heart does not pump enough oxygenated blood to the body. N-N intervals are essential to measuring some characteristics of tachycardia and bradycardia. For example, one of the important parameters in determining if a heart attack has occurred is the SDANN (standard deviation of the averaged N-N intervals) [21].
  To validate the accuracy of the HR prototype, ten measurements were obtained from ten patients by a nurse using a digital watch. The same action was then performed using the HR prototype. As shown in Table 3, the results obtained were very similar, verifying that the prototype performed correctly.

#### 3.1.2. Smart Monitoring

The SMTRPM performs smart monitoring of both the BT and HR variables through algorithms programmed into the Arduino Uno platform. The algorithms determine the patient's condition in real time based on the two variables. The smart monitoring system thus implements basic medical knowledge in the form of algorithms to diagnose conditions related to the BT and HR variables [16,20].

**Figure 5.**
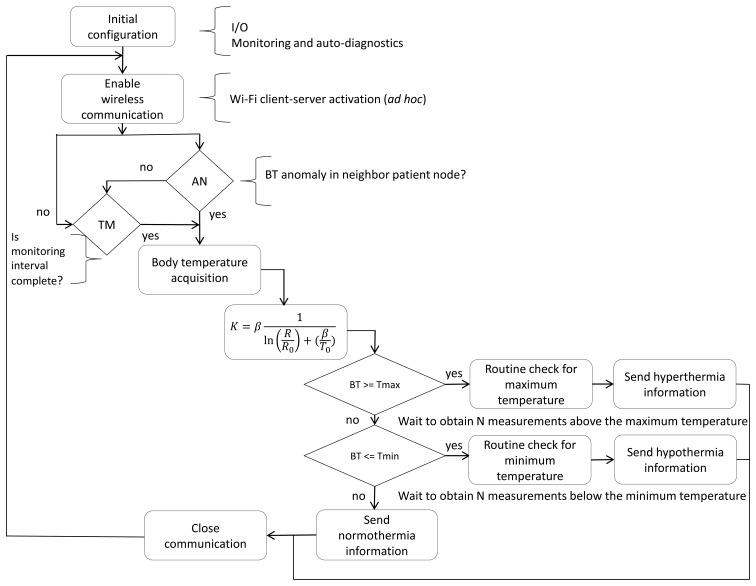
Flow diagram for the BT algorithm.

Figure 5 shows the flow diagram for the BT algorithm that runs locally on each patient node. It can be observed that the monitoring process can be initiated in two ways. The first is based on the BT monitoring interval established by the nurse. The second occurs when another patient node in the network detects a BT anomaly. When a WiFi communication from another node initiates the acquisition of the BT from the patient, ten BT samples are collected to verify whether the data consistently indicate the patient's condition to be hyperthermia, hypothermia, or normothermia. Finally, the patient node sends the BT and its classification to the last patient node. The last node sends the packet of information to the nurse server and the smartphone application.

**Figure 6.**
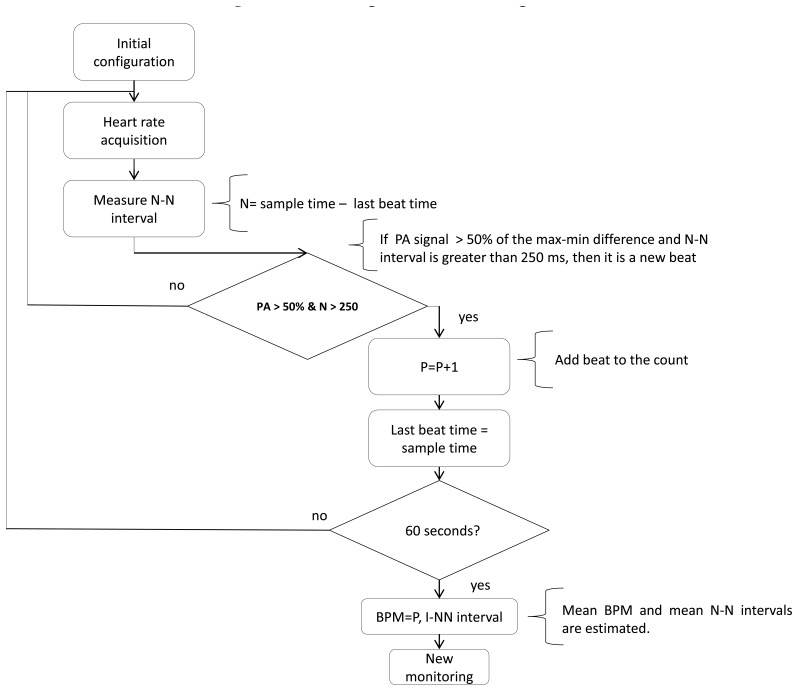
Flow diagram for the HR algorithm.

The HR smart monitoring process uses the algorithm developed by Jadav *et al.* [20] to determine the patient's condition. Figure 6 shows the flow diagram for the algorithm used to identify the occurrence of a heartbeat. The algorithm identifies the time at which the pulse reaches 50% of the difference between the maximum and minimum of the R wave. Data are obtained from the photoplethysmographic sensor every 2 ms using an interrupt routine. The N-N interval must be greater than 250 ms to be considered a true N-N event. When the system identifies a beat, it stores the elapsed time to establish a time reference for the next beat, and the N-N interval is estimated using the elapsed time between two beats. Every 60 s the system computes the average of the N-N intervals and the BPM. After obtaining the HR, the system identifies a tachycardic or bradycardic condition using a comparison process similar to the BT algorithm.

### 3.2. Network Structures

To implement the wireless monitoring system, the patient nodes of the SMTRPM transfer biomedical data through a WiFi network using a WiFly shield. The WiFly shield is a new device that converts universal asynchronous receiver/transmitter (UART) communication to WiFi (802.11 b/g) communication using the Roving Networks RN-131 module. The SMTRPM uses two network configurations, an *ad hoc* mode for communication with the nurse central server and an Internet mode for communication with the nurse's mobile Android smartphone, both using the TCP/IP protocol.

#### 3.2.1. *Ad Hoc* Mode

**Figure 7.**
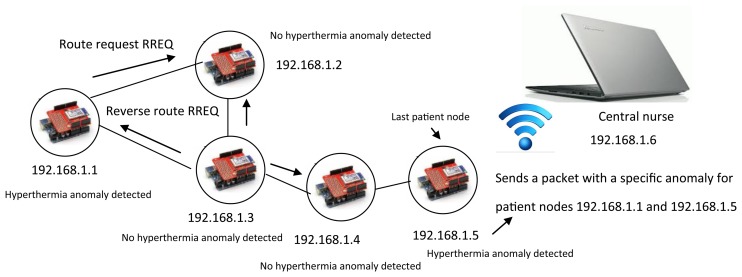
Specific anomaly detection via AODV (*ad hoc* on-demand vector) routing.

**Figure 8.**
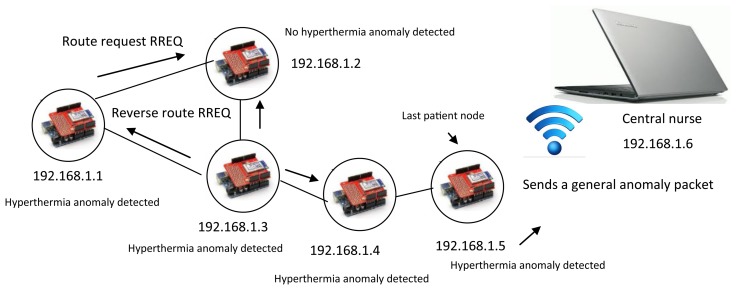
General anomaly detection via AODV routing.

The patient nodes and the nurse server are connected via a local *ad hoc* remote wireless network. The dynamic nature of this type of network requires intelligence in each of the network nodes for the delivery of information packets and for routing analysis [23]. Although the patient nodes have little mobility, they must find routes to deliver information. The patient nodes must determine if a specific or general anomaly has occurred in the room by sharing individual diagnostic information with the last patient node. When a patient presents an anomaly, the node sends the information to the last patient node and sends an alarm to the neighbor. This alarm causes the neighbor patient node to begin its own monitor process, and both patient nodes send the information to the last patient node, which is the only sink that obtains information from all the patient nodes. A reactive AODV (*ad hoc* on-demand vector) protocol is used to determine routing and anomaly conditions. The AODV algorithm determines a destination sequence number to evaluate a destination route [24]. The SMTRPM was tested with five patient nodes per room. Figures 7 and 8 show examples of specific and general anomaly detection, respectively.

In Figure 7, only two patient nodes have detected a hyperthermia anomaly. This information is sent to the last patient node via AODV routing. In the example, the last patient node determines that a specific hyperthermia anomaly has occurred because the SMTRP is programmed to identify a general anomaly only when three or more patients present the same anomaly, as in Figure 8.

The system adopts a hierarchical structure whereby there is a last patient node in each room. The last patient node is also a patient node with a program routine to create HR and BT package. Then, the last patient node sends the package to the nurse server. Table 4 shows an example of the structure of the HR package with specific anomalies at patients D and E.

#### 3.2.2. Internet Mode

Once the communication with the central nurse server is completed, the last patient node can connect to the Internet to send a diagnostic packet to a mobile device using an Android notification application. The mobile device application provides an alternative alert about the patient's health to the nurse even if the nurse is not at the central nursing station. The SMTRPM is implemented on a smartphone running Android OS 4.3.2. The mobile device and the last patient node are connected to the Internet. It is important that the network service company provide a static IP address to the PHI to avoid changes in the IP configurations of the last patient node and the mobile device from their original addresses.

### 3.3. Software System

The SMTRPM software system is the interface between the patient information and the nurse staff. The software system performs several activities to support the nurses, such as:

- displaying patient information in real time and updating the patient's condition when a hospitalized patient has been subjected to any treatment;
- generating a remote alarm when anomalies occur on a hospitalized patient;
- creating and using virtual files containing patient information.

#### 3.3.1. Nurse Server Interface (NSI)

**Figure 9.**
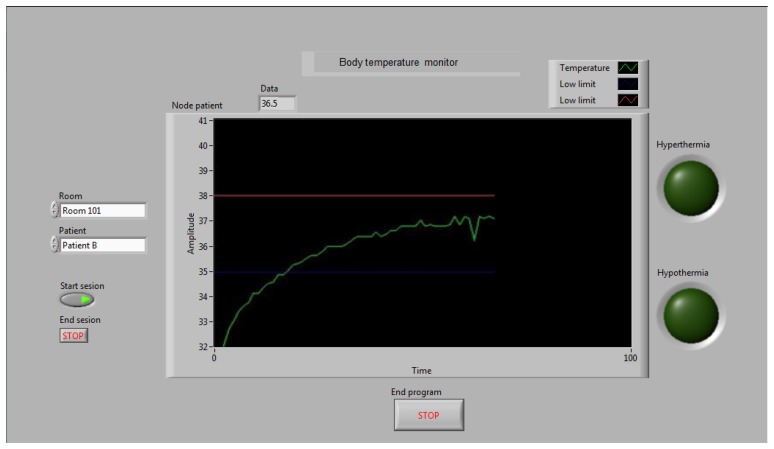
Monitor screen of the NSI showing the BT behavior of the last patient node measured in °C.

The NSI is a prototype that was created using LabView to support the analysis that the nursing staff performs to update patient health information. Typically, doctors give care instructions to the nurses when a patient has undergone treatment. For example, if a patient with an infection develops hyperthermia, the nurse dispenses medicine to reduce the patient's BT. Consequently, it is necessary to measure the BT frequently to determine the patient's rate of reaction to the treatment. Using the NSI, the nurse has the ability to remotely monitor the BT and HR at any time, reducing the time needed to monitor the patient and avoiding the need for continuous movement between rooms, especially when a nurse is in charge of many patients. Figure 9 shows the monitor screen of the NSI displaying a BT measurement.

**Figure 10.**
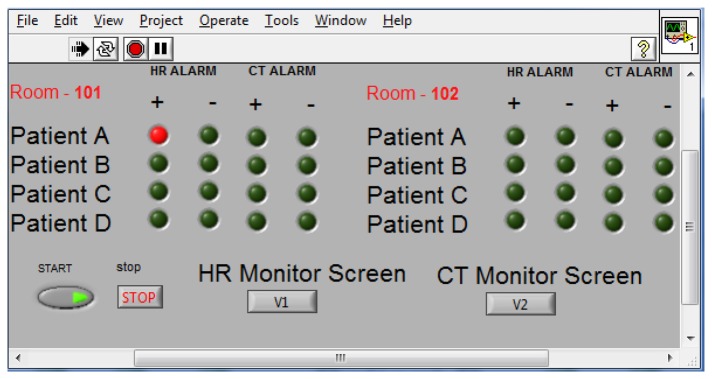
Main screen of the NSI showing the monitoring of two rooms and four patients.

The NSI runs on a PC at the nursing station. The application displays a waveform chart of the BT and HR variables on the monitor screen. The waveform chart can be used to support the nurse's analysis of the evolution of a patient's health or the reaction of a patient who has undergone treatment. In some cases, a patient may develop an allergy or rejection related to treatment, and then it becomes necessary to stop treatment. In those cases, the NSI can identify the patient's condition to the nurse faster than can remote alarms. Figure 10 shows the main screen of the NSI; the beta version is designed for two rooms with four patients in each room. When a patient develops an anomaly, the nurse can identify in which room the event occurred, the type of anomaly, and the patient with the anomaly. Then, the nurse can select the room and the patient on the monitor screen to observe the behavior of the specific variable associated with the anomaly because this information is associated with the IP address of the patient node.

#### 3.3.2. Mobile Nurse Monitor (Data Interoperability)

**Figure 11.**
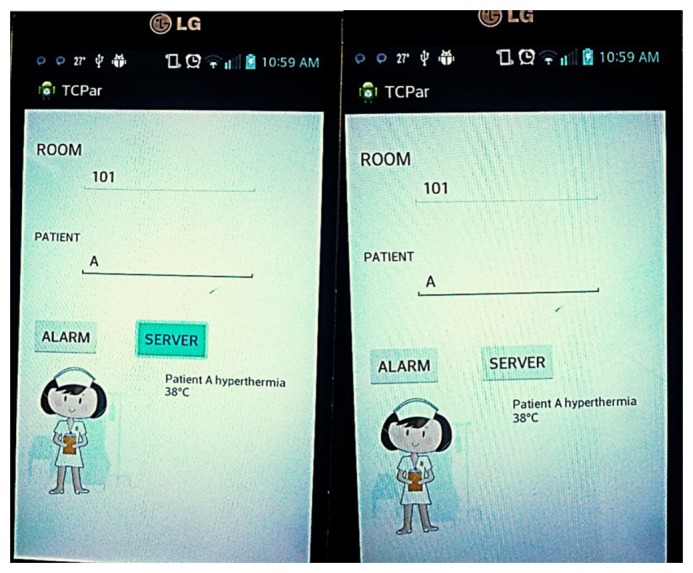
Patient hyperthermia condition alarm displayed on the mobile nurse monitor.

In recent years, advances in communications technology have enabled data sharing between two or more systems. Data interoperability allows the user to be informed of, and updated on, what is occurring in databases, files, or devices inside a network in specific situations [25]. The SMTRPM uses data interoperability to share information about hospitalized patients on a managed *ad hoc* WSN. The mobile nurse monitor (MNM) was developed in an Eclipse environment using the Android SDK (specifically, the ADT bundle for Windows) to provide continuous remote monitoring. The MNM provides the ability to test communication with individual clients via the TCP/IP protocol; however, the main objective was to create a mobile node that behaves in a server mode awaiting an alarm from the last patient node. Figure 11 shows the visual interface of the MNM application.

When an anomaly occurs at a patient node, the last patient node immediately sends a real-time alarm to the MNM application through the Internet mode. The application has the ability to inform the nurse about the alarm condition, identifying which patient node presented the anomaly and which type of anomaly was diagnosed. Because the nurse is notified anywhere inside the hospital, the MNM application can save valuable response time. The MNM application is particularly useful when the nurse is not at the central nursing station.

#### 3.3.3. Virtual Patient File (Augmented reality)

Frequently, nurses and doctors use handwritten patient files to view and update a patient's health status. The virtual patient file (VPF) application was developed to offer an alternative for the digital management of this information. Several technologies were evaluated for the design and implementation of the VPF. The options reviewed included near field communication (NFC), quick response (QR) codes and augmented reality (AR).

NFC and AR are considered to be among the most promising emerging mobile technologies. NFC can provide a good means of data tracking to prevent the possibility of human error when a doctor or nurse visits a patient, such as forgetting to note in a log that the patient was visited. However, given that proximity to the device is required for NFC, NFC cannot always serve as a substitute for AR or QR codes, which can both operate over a considerable distance. Today, many companies refuse to use NFC. Another major risk of NFC is computer hacking or phone hacking. Finally, NFC is relatively new, and not every mobile phone is compatible with this technology [26,27]. Because of these concerns, NFC was not further considered for the VPF.

QR codes are used to transfer information from a transitory medium to a cell phone. Once a QR code has been transferred to a cell phone, it may, for example, provide details about the business that provided the code, show a URL that can be clicked to watch the trailer for a movie, or issue a coupon that can be used at a local outlet. QR codes are more useful than standard barcodes because they can store much more data, including URL links, geographic coordinates, and text. However, one of their main disadvantages is people's lack of familiarity with QR codes. Another major disadvantage is the heterogeneity of mobile devices, which must be equipped with a camera and the correct reader software [28,29]. Unfortunately, many users whose mobile phones have cameras are unable to obtain QR reading software for their phones. For these reasons, QR codes were not further considered for the VPF.

AR is changing the way that people access information in their environment. The main goal of AR is to “augment everything everywhere for everyone”. The use of AR in conjunction with mixed reality (MR) breaks the barriers between virtual media, the physical world and our imagination by enriching our ability to interact with all three. AR could literally change the way we see the world, instantly providing information on any topic we choose to study [30]. The layering of information over a 3D space produces a new experience of the world. AR is fueling the broader migration of computing from desktops to mobile devices, bringing with it new expectations regarding access to information and new opportunities for learning [31]. Unlike QR codes, a key characteristic of AR is its ability to respond to user input. Additionally, AR is transforming the medicine and healthcare sectors significantly. In the domains of medicine and healthcare, AR not only can help save lives but also can help healthcare organizations make their existing processes more precise and efficient. Consequently, AR was selected for the implementation of the VPF.

The VPF was developed to offer an alternative for the management of digital information. The VPF is an MAR environment developed under the DroidAR framework [32]. DroidAR allows, among other AR features, marker-based tracking and the superposition of 2D and 3D models.

The VPF enables nurses to interact with digital information embedded within the physical environment and can be used to display information that otherwise would not be perceived by the regular human senses [33,34]. There are two forms of AR currently available: marker-based AR and markerless AR. For this research, marker-based AR was selected.

The VPF application uses two markers to display the BT and HR variables in real time. Each variable is associated with a marker ID. Both markers are pasted on the patient's headboard. The MAR application requires the following stages. Initially, a camera calibration process is performed in which the fundamental parameters of the camera are determined. These parameters determine where a 3D point is projected onto a 2D point (*i.e*., a pixel).

In an ideal camera, a 3D point (*X*, *Y*, *Z*) in space will be projected onto a pixel according to Equation (2).

(2) $$\begin{array}{cc} x=X\frac{fx}{Z}+cx; & y=Y\frac{fy}{Z}+cy \end{array}$$

where (*fx, fy*) is the focal length of the camera lens in the two dimensions and (*cx, cy*) represents the optical center of the sensor; both values are expressed in pixels. DroidAR uses a camera calibration method similar to that proposed in ARUCO [35].

After the camera calibration process has been performed, the system is ready to use. For example, when the nurse approaches a bed, he or she executes the application. Instantly, live video from the smartphone camera is acquired, and the nurse points to the markers. Then, the application performs the following computer vision algorithms. The images from the video stream are converted to binary images by a simple thresholding process described by Equation (3) [36].

(3) $$g(i,j)=\{\begin{array}{cc} 0, & f(i,j)\leq P\\ 1, & f(i,j)>P \end{array}$$

where the function *f* (*i, j*) is the input image (*i.e.*, the brightness of the pixels), *P* is the threshold and *g*(*i, j*) is the processed image.

If there is a marker in the scene, it is identified. Next, the positions and orientations of the markers relative to the camera coordinates are calculated. The symbol inside the marker is matched with templates, for which the only two possibilities are the marker for BT and the marker for HR. If a different figure is observed, it is discarded and the streaming continues. Finally, the position and orientation of the marker are computed. This is performed to align the virtual object with the marker. If the VPF detects the BT marker, a 2D thermometer image is superimposed onto the real scene in the position and orientation defined by the marker. In the case of the HR marker, a 2D heart image is superimposed.

**Figure 12.**
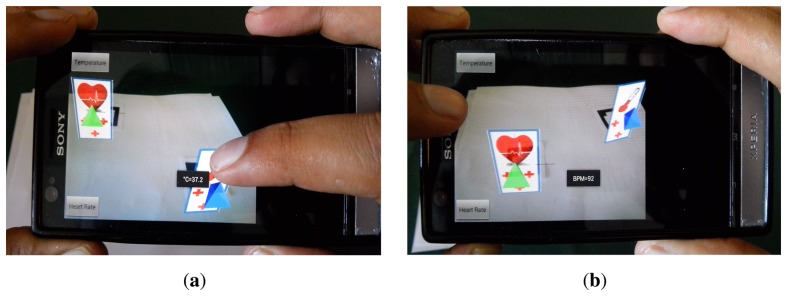
Example of VPF application: (**a**) VPF shows current BT value, and (**b**) VPF shows current HR value.

Additionally, there are two buttons (one at the top left and the other at the bottom left) in the application display used to request the status of a variable at any time. A touch event was programmed into the 2D images. Using this event, the nurse obtains the variable status in real time by touching its 2D image. Figure 12 shows the VPF application running.

All the data obtained are stored in a database to maintain information about each patient. Because the VPF application was developed with a commercial license for DroidAR, there are some limits of the AR environment. For example, only five markers can be detected in the same scene, and the VPF application cannot handle occlusion.

## 4. SMTRPM Evaluation

Several experiments were developed to test the main capabilities of the SMTRPM prototype. In the first level of the system, it is important to determine the response time for the acquisition of BT and HR signals. In the case of the BT response time, several measurements were performed using different values of *β*. Temperature behavior is often represented as a first order system in which *τ* is the time constant and k is the steady-state gain. The first order system is described by Equation (4).

(4) (τ)(y(t))+y(t)=kΔx

**Figure 13.**
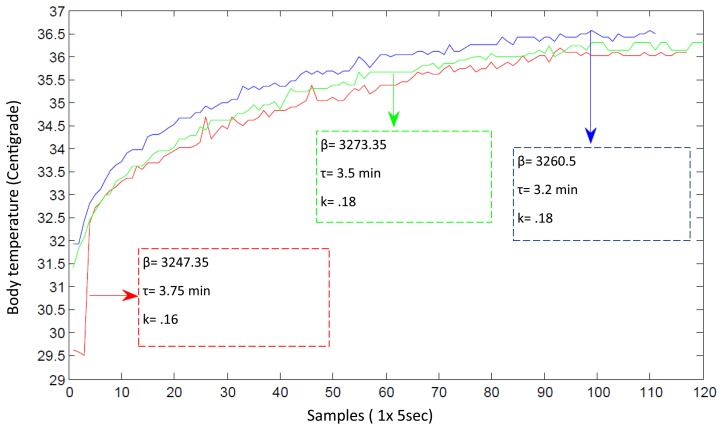
BT measurement trials with different *β* values to determine the response time of the BT prototype.

The plot in Figure 13 shows that the BT behaves as a first order system with an initial small delay. After several measurement trials, we found that the SMTRPM needs at least ten minutes to acquire an accurate value for the BT. This is not a disadvantage because it is generally recommended to wait three to five minutes to obtain an accurate BT value from commercial thermometers. Although the SMTRPM exceeds the acquisition time of commercial thermometers, it is important to remember that the system was developed for continuous monitoring, which means that ten minutes have passed before the first measurement is performed after the setup of the system. The nurse can update the BT of a patient whenever necessary after approximately ten minutes, without any additional waiting time.

**Figure 14.**
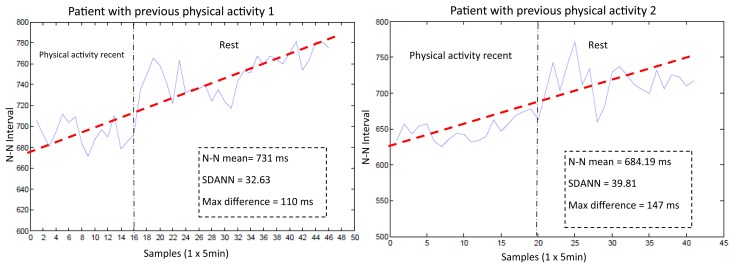
Tests of HR measurements in patients following physical activity.

Because the HR response time depends on the BPM, it is necessary to wait at least one minute to obtain the first sample. The measurement of the standard deviation of the averaged N-N intervals (SDANN) must be allowed sufficient time, preferably 24 h [37]. To determine the SDANN and the maximum change in the N-N intervals, the SMTRPM was tested in healthy patients with and without previous physical activity. The results obtained from the HR variability test are shown in Figure 14.

**Figure 15.**
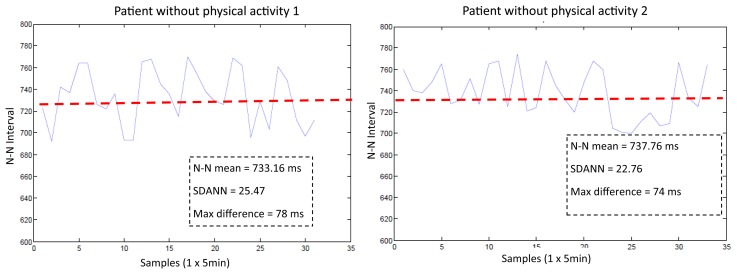
Tests of HR measurements in patients without previous physical activity.

The SMTRPM results show that after physical activity, the patients initially have a faster HR. However, after rest, the HR and the N-N intervals exhibit stable behavior, as shown in Figure 14, in which the red dotted line shows the trend in two patients after previous physical activity. Figure 15 shows results from two patients who did not engage in previous physical activity. The HR and the N-N intervals are stable throughout the test period. The red dotted line shows the mean of the N-N intervals. The results show that the SMTRPM could be used to measure the HR variability parameters to alert heart attacks or other HR disorders.

Additionally, it is important to determine the maximum distance over which a patient node can transmit the BT or HR information and the type of Internet network supported by the Android application. Data transmission from a patient node was tested over different lengths in the *ad hoc* mode. Additionally, the last patient node was tested over a private network and a public Internet network. Table 5 shows the results obtained from both tests.

As shown in Table 5, in the *ad hoc* mode, the patient node is able to transmit data within a perimeter of twenty meters without an antenna. If any room is farther than that distance, it is necessary to include a wireless antenna to perform data transmission. In the case of the Internet mode, the SMTRPM was tested on a public Internet network, and any electronic devices such as computers, smartphones, and iPods could be on the network without authorization. Most of the time, the public network was congested, and for this reason it was difficult for the SMTRPM to establish communication. The SMTRPM was also tested on private networks that required a web key (WSK2WAP) to access the Internet. On the private Internet networks, the SMTRPM established communication without problems, obtaining better performance by the Android application. Considering these results, it is important to connect the mobile device and the last patient node to a private Internet network to obtain better data transmission performance.

Finally, the SMTRPM was tested for diagnosing basic conditions related to the BT and HR variables by comparing 50 diagnoses both generated by the system and provided by nurses. To determine the reliability of SMTRPM diagnostics, Table 6 shows the false acceptance and false rejection rates that were found.

The results of the comparative test show that the SMTRPM provided correct BT diagnoses in most of the cases. However, for the HR conditions, there was a small rate of false diagnoses. One of the main reasons for this was that the HR sensor prototype was often not stably fixed to the patient's thumb. Consequently, a restraint mechanism should be developed for the final product design to obtain fewer false diagnoses.

## 5. Conclusions

In this paper, a smart multi-level tool for real-time remote patient monitoring (SMTRPM) was presented, which integrates two cutting-edge technologies, *i.e.*, WSN and MAR. To support nurse activities, the SMTRPM generates digital reports on patients' health. These reports are currently focused on body temperature and heart rate measurement. Moreover, the reports can be transmitted remotely within a communication distance of twenty meters without an antenna. The SMTRPM offers the nursing staff multiple ways to be informed when a patient presents an anomaly; updated patient vital signs can be obtained from the central nurse server interface or from the mobile nurse monitor.

During this research, it was observed that nurses must characterize a patient's health before and after medical treatment, surgery, or examinations. In countries where the government provides public health institutions (PHIs), there is an overpopulation of patients who require hospitalization. In most cases, nurses do not have access to new technology that could support activities such as patient monitoring, and instead rely on handwritten reports. The increasing occupancy rate of PHIs affects the functioning of the nursing staff because when a patient is admitted to a PHI, the nurses must be in continuous contact with the patient until the doctor provides a hospital discharge report. Consequently, the SMTRPM is presented as an alternative to improve nursing operations to achieve the following improvements:

- A decrease in the time needed to monitor patients, through characterizing a patient's health in real time.
- Automatic diagnosis in real time of conditions such as hyperthermia, hypothermia, tachycardia, and bradycardia.
- Remote alarm generation based on early detection of anomalies in a patient's health condition, avoiding the potentially serious consequences of late detection.
- Generation of virtual files for the viewing of patient health variables, avoiding the use of handwritten reports.

Finally, the project began as basic research to develop a new understanding of the foundations underlying WSN and MAR. However, the project evolved until it produced an important application for medical services. The primary aim was to understand the fundamental basis of an ultimate application. The top ten universities in the world are designing the newest technologies. Academic research is closely related to industrial technologies, and universities produce knowledge that is applicable outside of the academic setting. This research is an example of how to speed the translation of research into practice. Oftentimes, a gap exists between those who create the evidence base and those who are positioned to implement the research findings. The best way to obtain knowledge is through research and its application in real-life projects. Some of the planned future work includes the final product design of the sensors with manufacturing specifications, the implementation of databases in the cloud, and the integration of other variables (*i.e.*, sensors) to complement the basic diagnostics.

## Figures and Tables

**Table 1.** Research on biomedical wireless monitoring systems.

**Author**	**Application**	**Variables**	**Communication**	**Sensors**	**Monitors**	**Benefits**
Dağtas *et al.* [10], 2007	Patient monitoring in a smart home	Cardiac activity	ZigBee	ECG	CPU	Communication with home server via ZigBee; incoming data stored for future reference
Curtis *et al.* [3], 2008	Ambulatory patient monitoring system	Vital signs, position of patient	WiFi	GPS, ECG, accelerometer	PDA, CPU	Open platform, low cost, geo-positioning	
Abbate *et al.* [11], 2010	Monitoring system for the elderly	Acceleration and tilt angle	ZigBee, Bluetooth	Accelerometers, gyroscope	CPU	Mobility of nodes
Gómez *et al.* [12], 2011	Cardiac condition monitoring system	Cardiac activity, pressure and volume	ZigBee	Conductance catheter	FPGA, CPU	Management of multiple signals
Cancela *et al.* [13], 2011	Parkinson's disease monitoring system	Gait, posture, leg and hand movement	ZigBee	Accelerometer, gyroscope, microphone	PDA	Focused on one disease
Vijayalakshmi *et al.* [14], 2012	General patient monitoring system	Cardiac activity, respiration, muscle activity	ZigBee, Bluetooth, WiFi	EMG, EEG, EKG	PDA, CPU	Communication with medical server through Internet
Our proposal, 2014	General patient monitoring system	Cardiac activity, Body temperature	WiFi	ECG, Body temperature	Smartphone, CPU	Communication with nurse server and smartphones through Internet

**Table 2.** Comparative trials between the BT prototype and the commercial thermometer.

**Beta (***β***)**	**BT prototype Mean Temperature**	**Commercial Thermometers Mean Temperature**	**Mean Relative Error**
3247.35	36.05 °C	36.5 °C	0.012
3273.35	36.16 °C	36.5 °C	0.009
**3260.5**	36.43 °C	36.5 °C	0.002

**Table 3.** Comparative trials between the HR prototype and a conventional digital watch.

**Patient**	**Manual Measurement (BPM)**	**HR Prototype Measurement (BPM)**	**Mean Relative Error**
1	72	76	0.056
2	65	65	0
3	70	68	0.029
4	72	76	0.056	
5	68	65	0.044	
6	65	69	0.062
7	72	68	0.056
8	82	85	0.037	
9	86	85	0.012	
10	65	65	0

**Table 4.** HR package.

**Condition**	**PA**	**PB**	**PC**	**PD**	**PE**
Normal	1	1	1	0	0
Tachycardia	0	0	0	1	0
Bradycardia	0	0	0	0	1

**Table 5.** Communication test (packet loss rate values).

**Transmission Distance in *Ad Hoc* Network**	**Packet Loss Rate**	**Internet Network**	**Packet Loss Rate**
6 m	0.13%	Private one	0.11%
12 m	0.19%	Private two	0.14%
20 m	0.8%	Public one	48%
23 m	No comm	Public two	No comm

**Table 6.** Comparative test between the diagnostics provided by the SMTRPM and the nurses.

**Condition**	**Detected Parameter**	**False Rejection Diagnosis (FRD)**	**False Acceptance Diagnosis (FAD)**
Tachycardia	*HR* > 100 *BPM*	4%	6%
Bradycardia	*HR* < 60 *BPM*	2%	10%
Hyperthermia	*CT* > 37.5 °*C*	0%	0%
Hypothermia	*CT* < 36.0 °*C*	2%	0%

## References

[1] (2010). World Health Organization Health Workforce, Infrastructure and Essential Medicines. World Health Statistics 2010.

[2] Callen J., Hordern A., Gibson K., Li L., Hains I., Westbrook J. (2013). Can Technology Change the Work of Nurses? Evaluation of a Drug Monitoring System for Ambulatory Chronic Disease Patients. Int. J. Med. Inf..

[3] Curtis D., Pino E., Bailey J., Shih E., Waterman J., Vinterbo S., Stair T., Guttag J., Gree R., Ohno-Machado L. (2008). SMART—An Integrated Wireless System for Monitoring Unattended Patients. J. Am. Med. Inf. Assoc..

[4] Wang D., Wang P. (2014). Understanding Security Failures of Two-Factor Authentication Schemes for Real-Time Applications in Hierarchical Wireless Sensor Networks. Ad Hoc Netw..

[5] Kim Y., Lee S. (2014). Energy-Efficient Wireless Hospital Sensor Networking for Remote Patient Monitoring. Inf. Sci..

[6] Oliveira M., Fernandes M., Primo J., Reis H., Nicola P. (2013). Remote Monitoring *Versus* Conventional Follow-up for Implantable Cardiac Devices: Rationale and Design of the PORTLink (PORTuguese Research on Telemonitoring with CareLink) trial. Port. J. Cardiol..

[7] He Z., Xu W., Liu G. Design of a Wireless Medical Monitoring System.

[8] Fensli R., Gundersen T., Snaprud T., Hejlesen O. (2013). Clinical Evaluation of a Wireless ECG Sensor System for Arrhythmia Diagnostic Purposes. J. Biomed. Eng..

[9] Misra S., Chatterjee S. (2014). Social Choice Considerations in Cloud-Assisted WBAN Architecture for Post-Disaster Healthcare: Data Aggregation and Channelization. Inf. Sci..

[10] Dagtas S., Pekhteryev G., Sahinoglu Z. Multi-Stage Real Time Health Monitoring via ZigBee in Smart Homes.

[11] Abbate S., Avvenuti M., Corsini P., Light J., Vecchio A. (2010). Monitoring of Human Movements for Fall Detection and Activities Recognition in Elderly Care Using Wireless Sensor Network: A Survey. Wireless Sensor Networks: Application—Centric Design.

[12] Gómez M., Goy C., Bolognini P., Herrera M. (2011). FPGA Implementation of a ZigBee Wireless Network Control Interface to Transmit Biomedical Signals. J. Phys. Conf. Series.

[13] Cancela J., Pastorino M., Arredondo M., Pansera M., Pastor-Sanz L., Villagra F., Pastor M., Gonzalez A. Gait Assessment in Parkinsons Disease Patients through a Network of Wearable Accelerometers in Unsupervised Environments.

[14] Vijayalakshmi B., Ram Kumar C. Patient Monitoring System Using Wireless Sensor based Mesh Network.

[15] Srividyadevi P., Pusphalatha D., Sharma P. (2013). Measurement of Power and Energy Using Arduino. Res. J. Eng. Sci..

[16] Panagiotis K., Poulopoulou M., Papahatzi A., Souleles P. (2005). Effects of Hypothermia and Shivering on Standard PACU Monitoring of Patients. J. Am. Assoc. Nurse Anesth..

[17] Dongale T., Kamat R. (2013). Modelling of NTC Thermistor Using an Artificial Neural Network for Non- Linearity Compensation. Inf. Eng. Int. J..

[18] Kranjec J., Begus S., Gersak G., Drnovsek J. (2014). Non-Contact Heart Rate and Heart Rate Variability Measurements: A Review. J. Biomed. Signal Process. Control.

[19] Pancar E., Yuksel S., Yenercag M., Soylu K., Aydin F., Senturk N., Yucel H., Canturk T., Turanli A. (2014). Impaired Heart Rate Recovery Indices in Psoriasis Patients. J. Med. Sci. Monit..

[20] Jadav V.H., Sitapra H.J. (2013). A Novel Approach for Designing of Pulse Transit Time Measurement Device for Physiological Applications. J. Inf..

[21] Buttá C., Tuttolomondo A., Raimondo D., Milio G., Miceli S., Attanzio M., Giarrusso L., Licata G., Pinto A. (2013). The Supraventricular Tachycardias: Proposal of a Diagnostic Algorithm for the Narrow Complex Tachycardias. J. Cardiol..

[22] Dias I., Miguel M., Galvao P., Cerqueira J., Machado Y., Antônio L., Pinheiro N., Nascimento P., Amorim R., Rodrigues G. (2011). Retrospective Analysis of Risk Factors and Predictors of Intraoperative Complications in Neuraxial Blocks at Faculdade de Medicina de Botucatu-UNESP. Braz. J. Anesthesiol..

[23] Lee C., Jeong T. (2011). FRCA: A Fuzzy Relevance-Based Cluster Head Selection Algorithm for Wireless Mobile *Ad Hoc* Sensor Networks. Sensors.

[24] Alhosban A., Ababneh I., Malik Z. (2012). GFDA: Route Discovery Algorithms for On-Demand Mobile Ad Hoc Routing Protocols. J. Procedia Comput. Sci..

[25] Wajahat K., Maqbool H., Khalid L., Muhammad A., Farooq A., Sungyoung L. (2013). Process Interoperability in Healthcare Systems with Dinamyc Semantic Web Services. Computing.

[26] Brolla G., Vodickab E., Boringc S. (2013). Exploring Multi-User Interactions with Dynamic NFC-Displays. Pervasive Mob. Comput..

[27] Prinz A., Menschner P., Leimeister J. (2012). Electronic Data Capture in Healthcare-NFC as Easy Way for Self-Reported Health Status Information. Health Policy Technol..

[28] Shin D., Jung J., Chang B. (2012). The psychology behind QR codes: User experience perspective. Comput. Hum. Behav..

[29] Garateguy G., Arce G., Lau D., Villarreal O. (2014). QR Images: Optimized Image Embedding in QR Codes. IEEE Trans. Image Process..

[30] Kahn S. (2013). Reducing the Gap between Augmented Reality and 3D Modeling with Real-Time Depth Imaging. Virtual Real..

[31] Escobedo L., Tentori M., Quintana E., Favela J., Garcia-Rosas D. (2014). Using Augmented Reality to Help Children with Autism Stay Focused. IEEE Pervasive Comput..

[32] Kounavis C., Kasimati A., Zamani E. (2012). Enhancing the Tourism Experience through Mobile Augmented Reality: Challenges and Prospects. Int. J. Eng. Bus. Manag..

[33] Dunleavy M. (2014). Design Principles for Augmented Reality Learning. Techtrends.

[34] Caia S., Wanga X., Chiang F. (2014). A Case Study of Augmented Reality Simulation System Application in a Chemistry Course. Comput. Hum. Behav..

[35] Garrido S., Munoz R., Madrid F., Marin M. (2014). Automatic Generation and Detection of Highly Reliable Fiducial Markers Under Oclusion. Pattern Recognit..

[36] Prochazka D., Koubek T. (2011). Augmented Reality Implementation Methods in Mainstream Applications. Acta Univ. Agric. Silvic. Mendel. Bronensis.

[37] Zulfiqar U., Jurivich D., Gao W., Singer D. (2010). Relation of High Heart Rate Variability to Healthy Longevity. Am. J. Cardiol..

